# End-user perceptions of a patient- and family-centred intervention to improve nutrition intake among oncology patients: a descriptive qualitative analysis

**DOI:** 10.1186/s40795-020-00353-8

**Published:** 2020-07-21

**Authors:** Andrea P. Marshall, Georgia Tobiano, Shelley Roberts, Elisabeth Isenring, Jasotha Sanmugarajah, Deborah Kiefer, Rachael Fulton, Hui Lin Cheng, Ki Fung To, Po Shan Ko, Yuk Fong Lam, Wang Lam, Alex Molassiotis

**Affiliations:** 1 Nursing and Midwifery Education and Research Unit, Gold Coast Health, 1 Hospital Blvd, Southport, QLD 4215 Australia; 2 Menzies Health Institute Queensland, Griffith University, Parklands Drive, Southport, QLD 4215 Australia; 3 School of Nursing and Midwifery, Griffith University, Gold Coast Campus Parklands Drive, Southport, QLD 4215 Australia; 4 Division of Allied Health, Gold Coast Health 1 Hospital Blvd, Southport, QLD 4215 Australia; 5 School of Allied Health, Griffith University, Parklands Drive, Southport, QLD 4215 Australia; 6 Nutrition & Dietetics, Faculty of Health Sciences & Medicine, Bond University Level 2, Bond Institute of Health and Sport, 2 Promethean Way, Robina, QLD 4226 Australia; 7 Medical Oncology, Gold Coast Health, 1 Hospital Blvd, Southport, QLD 4215 Australia; 8 School of Nursing, Faculty of Health and Social Sciences, Room A401, Chung Sze Yuen Building, The Hong Kong Polytechnic University, Hung Hom, Kowloon, Hong Kong; 9 Dietetics Department, Alice Ho Miu Ling Nethersole Hospital Hospital Authority, Chuen On Rd, Tai Po, Hong Kong; 10 Kowloon East Cluster, Hospital Authority, Hong Kong, China; 11 Department of Medicine, Haven of Hope Hospital, Haven of Hope Rd, 8, Tseung Kwan O, Hong Kong; 12 Dietetics Department, Haven of Hope Hospital, Haven of Hope Rd, 8, Tseung Kwan O, Hong Kong

**Keywords:** Cancer, Community healthcare, Medical oncology, Nutritional support, Outpatient clinics, Patient-centered care, Person-centred care, Qualitative research

## Abstract

**Background:**

People with cancer are at high risk of malnutrition. Nutrition education is an effective strategy to improve patient outcomes, however, little is known regarding the impact of family and/or carer involvement in nutrition education and requires investigation. The purpose of the study was to evaluate PIcNIC (**P**artnering with fam**i**lies to promote **n**utrition **i**n **c**ancer care) intervention acceptability from the perspective of patients, families and health care providers.

**Methods:**

A descriptive qualitative study was undertaken at an inpatient and an outpatient hospital setting in Australia and an outpatient/home setting in Hong Kong. A patient-and-family centred intervention including nutrition education, goals setting/nutrition plans, and food diaries, was delivered to patients and/or families in the inpatient, outpatient or home setting. Semi-structured interviews were used to explore perceptions of the intervention. 64 participants were interviewed; 20 patients, 15 family members, and 29 health care professionals. Data were analysed using deductive and inductive content analysis.

**Results:**

Two categories were identified; 1) ‘context and intervention acceptability’; and 2) ‘benefits of patient- and family-centred nutrition care’. Within each category redundant concepts were identified. For category 1 the redundant concepts were: the intervention works in outpatient settings, the food diary is easy but needs to be tailored, the information booklet is a good resource, and the intervention should be delivered by a dietitian, but could be delivered by a nurse. The redundant concepts for category 2 were: a personalised nutrition plan is required, patient and family involvement in the intervention is valued and the intervention has benefits for patients and families. Converging and diverging perceptions across participant groups and settings were identified.

**Conclusions:**

In this paper we have described an acceptable patient- and family-centred nutrition intervention, which may be effective in increasing patient and family engagement in nutrition care and may result in improved nutrition intakes. Our study highlights important contextual considerations for nutrition education; the outpatient and home setting are optimal for engaging patients and families in learning opportunities.

## Background

Patients undergoing treatment for cancer face a number of issues relating to nutrition. Common nutritional concerns include malnutrition (affecting 50–80% of cancer patients) and metabolic derangements that can occur due to the tumour and/or treatment [1]. Commonly reported nutrition-impacting symptoms include peculiar tastes, no appetite, early satiety, indigestion and nausea which can reduce dietary intakes and increase malnutrition risk [2]. Malnutrition is of concern as it is associated with increased morbidity, longer hospital length of stay (LOS), increased unscheduled re-admissions, poorer quality of life and reduced response and tolerance of treatment, culminating in decreased survival and increased healthcare costs [1].

The risk of malnutrition for people with cancer is high because both the disease and associated treatments can significantly compromise nutritional status. It is suggested that between 10 and 20% of patients with cancer may die because of malnutrition rather than the malignancy itself [3, 4]. Consequently, strategies to improve nutrition intakes for people with cancer are warranted. Nutrition counselling traditionally provided one-on-one by a dietitian to the patient is among the most commonly used interventions to support malnourished patients with cancer and has been shown to improve short and medium-term outcomes including nutrition intakes, nutritional status, and quality of life [5].

Patients and their family caregivers are interested in nutrition and wish to receive more information, especially with regards to nutritional supplements and tips to help manage side effects [2]. Patients with cancer may experience suboptimal care coordination throughout the trajectory of their care and hear mixed messages around nutrition in cancer care from healthcare providers, friends or family members and the media. This conflicting information and a lack of a continuum of care is likely to be confusing to patients, highlighting the need for timely and ongoing patient-focused nutrition care, with formal and informal support [6]. Family, friends and carers are an untapped workforce who are interested in participating in their patient’s care [7].

Our team developed and piloted a patient- and family-centred intervention for improving nutrition intake among patients receiving curative cancer treatment in hospital (Australia) and advanced cancer patients at home (Hong Kong). Patients (*n* = 53), family members (*n* = 22) and health professionals (*n* = 30) found the intervention helpful and acceptable, and patients and families indicated they would take part in a future similar study. Energy and protein intakes improved from baseline to end of intervention (mean increase of 22 to 26 kcal/kg/day and 0.9 to 1.0 g/kg/day respectively). The aim of this study was to evaluate intervention acceptability from the perspective of patients, families and health care providers in two different clinical settings in two countries.

## Methods

### Study overview

This descriptive qualitative study [8] was a component of a larger pilot study testing the acceptability of a patient and family-centred intervention for improving nutrition among oncology patients [9]. The study received ethical approval from the participating health services and university (Australian site: Gold Coast Health HREC/16/QGC/73 and Griffith University 2016/200; Hong Kong site: Hong Kong Polytechnic University KC/KE-16-0138/ER-2). Written informed consent was received from all participants after information about the researchers and purpose of the research was provided.

### Setting and sample

The study was conducted at two international sites; one in Australia and one in Hong Kong. The context of each setting differed. At the Australian site, the study was conducted in both an inpatient (oncology ward) and an outpatient (oncology clinic) setting, in a large metropolitan tertiary teaching hospital in southeast Queensland. In Hong Kong, the study was conducted in an outpatient palliative care/home setting; participants were recruited through a palliative care clinic of a local hospital in Kowloon. Participants in this qualitative sub-study included a sub-sample of patients, family members (including paid carers) and staff who were involved in the PIcNIC (**P**artnering with fam**i**lies to promote **n**utrition **i**n **c**ancer care) intervention across the two study sites. Purposive sampling was used to ensure a broad range of participants were represented from each group (patients, families and health care professionals) at each site. Participants were approached either in person or by telephone (Australia outpatients) to inform them of the study.

### The intervention

The intervention was a face-to-face education program (one session of approximately 30 min) delivered to the patient and/or their family member supported by a written booklet, including education of the importance of nutrition therapy during cancer, ways to support nutrition intake, approaches to manage nutrition impacting symptoms, and strategies for eating and weight related issues (see Additional File 1). At the Hong Kong site, goal-setting and components on psychosocial aspects of nutritional care were also included, mainly focusing on dealing with eating-and weight-related distress. Participants were also educated on food diaries and encouraged to monitor patients’ oral intake. This education was reinforced on hospital discharge (Australian inpatient setting) or 2–4 weeks post intervention delivery (Hong Kong setting). In the Australian setting a post-discharge nutrition plan was provided. Further details on the intervention can be found in the pilot study paper [9].

In Australia, the intervention was delivered in both the inpatient and outpatient context. For inpatients, the intervention was delivered to the patient, and family member if available, on the oncology ward by a dietitian. For outpatients, the intervention was delivered to the patient, and family member if available, in the oncology dietitian outpatient clinic. In Hong Kong, the intervention was delivered at the patient’s home to the patient and family or carer. The duration of the intervention varied. In the Australian inpatient setting the duration was typically 5–7 days, depending on the patient’s length of hospital stay. In the outpatient setting the intervention was delivered at the patient’s first outpatient appointment with follow-up 2 weeks thereafter. In Hong Kong patients and their families were followed up over a 4-week period.

### Standard care

At the Australian hospital, inpatient standard care included nutrition screening (using the Malnutrition Screening Tool) [10], and referral to a dietitian if the patient was considered at risk of malnutrition. The hospital had an electronic foodservice system and provided patient meals from a set, 2-week cyclic menu, which patients ordered through bedside computer screens. Oral nutrition supplements were prescribed by a dietitian if required. Outpatient referrals to either the radiation or general oncology dietitian clinics were done by any member of the health care team, usually for patients who had lost weight or who had nutrition impacting symptoms. In the outpatient department, patients have a nutritional assessment undertaken and are provided with tailored advice on managing nutrition intake and nutrition impacting symptoms.

In Hong Kong, patients attended medical appointments at an outpatient palliative care clinic where they were referred to a dietitian if patients had major nutritional symptoms, including reduced food intake, poor appetite and weight loss, as assessed and determined by physicians or nurses. Standard care included regular follow-ups and home visits by nurses when necessary. Details of participant inclusion and exclusion criteria and recruitment can be found in the pilot study paper [9].

### Data collection

Semi-structured interviews were used to explore patient, family and health care professional perceptions of participating in the PIcNIC intervention, focusing particularly on intervention acceptability. A semi-structured interview guide was used to ask participants about their experiences and perceptions of participating in the PIcNIC intervention. Table 1 provides examples of interview questions for patients, families and staff. Interviews were audio recorded on a handheld device and transcribed verbatim; transcribed data were supplemented by field notes and contact summaries [11]. Chinese transcripts were translated into English for analysis by a bilingual member of the research team. Data collection continued until data saturation was reached, that is, when no new codes emerged from the data.

**Table 1:** Example semi-structured interview questions

Patient and family member questions	Health care professional questions
Why did you choose to participate in the PICNIC study (the study)? How did you feel when you were approached to participate in the study?	To what extent do you believe patients and their family members should be advocating for best nutrition practice?
What did you think about the education session with the dietitian? Were you able to ask questions, and were these answered to your satisfaction? Did you feel you could adequately report on your/the patient’s nutrition history?	How do you see the role of the patient and their family in the context of cancer care? Do you think they should be actively involved in some aspects of patient care and decision making?
Was the information provided in the booklet clear? Was it useful/ relevant? Are you able to suggest any ways in which the booklet could be improved?	Do you think providing this level of nutrition information was beneficial for patients/families?
Were you involved in recording your/your family member’s food intake on the food record? If not, why not? If so, did you find this easy or difficult? Were there any advantages/ disadvantages to keeping the booklet? Why?	Did you notice whether patients or families were completing the food intake chart? Why or why not do you think they completed it? Can you see any barriers/facilitators to patients/ families completing the food chart? Do you think it’s beneficial for them to complete it? Can you see any ways of making the food chart completion easier for patients/families?
Did you ask health care staff questions about your/your family member’s nutrition? If yes, do you think staff were receptive to these questions? Did you feel comfortable having these conversations with staff?	Did patients or their family members make specific enquiries about nutrition? Do you think patients and families who participated in the study asked more questions of the staff than families that didn’t?
Patients: What did you think about involving your family in this intervention? Do you think your family member was a positive support in your nutrition? Did you have any issues with your family member(s) being involved? Family members: Do you think you could tell whether your family member was eating enough?	Do you think this intervention (i.e. session with the dietitian, asking patients/families to complete food charts, and encouraging patients/families to be active participants in their nutrition care) is feasible in real practice?
Overall, do you think this intervention helped with your/your family member’s nutrition? Why/why not? Would you participate in something like this again? Why/why not?	Can you comment on the intervention overall? Is there anything you would change?

In Hong Kong, face-to-face interviews were primarily conducted by a trained research assistant in Chinese with the patient and/or family member, either at the home or occasionally in the outpatient palliative care clinic. In Australia, interviews were conducted in English, via face-to-face interviews (GT) with inpatients; and via telephone (RF) with outpatients, by interviewers with research qualifications or training. All interviewers did not have a prior relationship with any interviewees. Health care professionals at both sites were interviewed in a staff room, at a time of mutual convenience.

### Data analysis

Content analysis was used to analyse transcribed interview data. During the initial reading of data, we discovered variation between patients/families and health care professionals in terms of intervention acceptability, and between Australia and Hong Kong in terms of patient-and-family centred aspects of the intervention. Thus, we used Benzer et al.’s [12] concepts to guide analysis, which are suitable to use when variation is found in qualitative research within and across sites and participant groups. Three concepts were used to guide analysis: 1) ‘redundant’ perceptions were conceptualised as perceptions completely shared across participant groups and sites; 2) ‘convergent’ perceptions were defined as instances when participants agreed with redundant concepts, but elaborated on these by demonstrating some variation in the phenomenon of interest; and 3) ‘divergent’ perceptions were ideas that were unique to redundant concepts [12].

An inductive and deductive content analysis approach was undertaken by two authors (GT, APM) [13]. Inductive content analysis occurred first, to allow overarching categories to be identified [13]. Transcripts were coded line-by-line, and similar codes were grouped together into redundant concepts based on codes representing shared perceptions. The redundant concepts were read many times, and flow diagrams were used to organise the concepts that were based on similar topics, allowing larger categories to be developed.

Next, a deductive content analysis approach was undertaken, allowing redundant concepts to be explored in more depth [13]. Tables were created for each redundant concept, and within each table six columns were created for each ‘group’; 1) Hong Kong health care professionals; 2) Hong Kong patients; 3) Hong Kong families; 4) Australian health care professionals; 5) Australian patients; and 6) Australian families. Data were recoded as convergent or divergent perceptions for each group.

The first author (APM) participated in frequent discussions with (GT) about the coding and grouping of data to enhance trustworthiness, and frequent discussions were held between all three of these authors to ensure codes, redundant concepts and categories adequately described the data to enhance credibility.

## Results

In total, 64 participants were interviewed; 35 participants were from Australia and 29 from Hong Kong. Data were obtained from 20 patients (Australia *n* = 13; Hong Kong *n* = 7), 15 family members (Australia *n* = 4; Hong Kong *n* = 11) and 29 health care professionals (Australia *n* = 18; Hong Kong n = 11) (Table 2). There was a 1:1 ratio of patients consenting and declining consent, owing to being too unwell to participate.

**Table 2:** Interview participants by country

Participant	Interview Type	Australia	Hong Kong
Staff	Individual	6 individual interviews were conducted with senior oncology health care professionals including 4 dietitians, 1 clinical nurse coordinator and 1 oncologist.	1 individual interview was conducted (senior medical director).
	Group	12 oncology nurses participated in one of two group interviews (n = 7 and n = 5).	2 group interviews conducted with a total of 10 participants (3 doctors, 2 dietitians, and 5 nurses).
Patients and family	Individual	A total of 13 patients were interviewed (5 inpatient and 8 outpatients). Four family members were interviewed.	A total of seven patients and 11 family members were interviewed.
	Group	No group interviews were conducted.	No group interviews were conducted.
Total duration of interviews (mins)		428	339

Participant characteristics can be seen in Table 3. Patients had a mean age of 63–72.4 years, and 57.1–62.9% were female. The most frequently reported cancer diagnosis in Australia was breast cancer, and in Hong Kong was lung cancer. The most frequent malnutrition screening score in Australia was 0 or 1, while in Hong Kong it was 2. For family participants, all Australian participants were female spouses/de facto partners. In Hong Kong, most participants were female, and had a range of relationships to the patients. For health care professionals, 70–94.4% of participants were female, and were most frequently nurses across both sites. Australian health care professionals had a range of clinical experience, where most Hong Kong participants had more than 15 years of clinical experience. In Australian similar numbers of participants had been involved with a patient or family in the PIcNIC intervention; this was not the case for most Hong Kong health care professionals.

**Table 3:** Characteristics of participants

**Patient participants**		
**Characteristics**	**Australia (n = 13)**	**Hong Kong (n = 7)**
**Age in years (mean/SD)**	63.0 (18)	72.4 (13.4)
**Gender n (%):**		
- Female	9 (62.9%)	4 (57.1%)
- Male	9 (30.8%)	3 (42.9%)
**Diagnosis n (%):**		
- Breast cancer	4 (30.8%)	0 (0.0%)
- Colorectal cancer	2 (15.4%)	2 (28.6%)
- Gastric cancer	1 (7.7%)	0 (0.0%)
- Kidney cancer	0 (0.0%)	1(14.3%)
- Lung cancer	0 (0.0%)	4 (57.1%)
- Oesophageal cancer	1 (7.7%)	0 (0.0%)
- Ovarian cancer	1 (7.7%)	0 (0.0%)
- Pancreatic cancer	3 (23.1%)	0 (0.0%)
- Skin cancer	1 (7.7%)	0 (0.0%)
**MST score n (%):**		
- 0	2 (40.0%)	0 (0.0%)
- 1	2 (40.0%)	0 (0.0%)
- 2	0 (0.0%)	6 (85.7%)
- 3	0 (0.0%)	1 (14.3%)
- N/A	1 (20.0%)	0 (0.0%)
**PG-SGA SF score (median/IQR)**	7 (5–12)	10(1–14)
**Family participants**		
**Characteristics**	**Australia (n = 4)**	**Hong Kong (n = 11)**
**Age in years n (%):**		
- < 21	0 (0.0%)	0 (0.0%)
- 21–30	1 (25.0%)	0 (0.0%)
- 31–40	0 (0.0%)	2 (18.2%)
- 41–50	0 (0.0%)	4 (36.4%)
- 51–60	0 (0.0%)	0 (0.0%)
- 61–65	1 (25.0%)	2 (18.2%)
- > 65	2 (50.0%)	2 (18.2%)
- Prefer not to respond	0 (0.0%)	1 (9.1%)
**Gender n (%):**		
- Female	4 (100.0%)	7 (63.6%)
- Male	0 (0.0%)	4 (36.4%)
**Relationship to patient n (%):**		
- Spouse/de factor partner	4 (100%)	3 (27.3%)
- Child	0 (0.0%)	6 (54.6%)
- Daughter in law	0 (0.0%)	1 (9.1%)
- Domestic helper	0 (0.0%)	1 (9.1%)
**Approximate combined household income per year in AUD n (%):**		
- 40,001-60,000	1 (25.0%)	N/A
- 100,001-120,000	1 (25.0%)	N/A
- Prefer not to respond	2 (50.0%)	N/A
**Approximate combined household income per month in HKD n (%):**		
- < 10,000	N/A	3 (27.3%)
- 15,000- 20,000	N/A	2 (18.2%)
- 25,00-30,000	N/A	1 (9.1%)
- Prefer not to respond	N/A	5 (45.5%)
**Highest level of education complete** **n (%):**		
- Elementary school	0 (0.0%)	2 (18.2%)
- Middle school	0 (0.0%)	4 (36.4%)
- Some high school	1 (25.0%)	3 (27.3%)
- Some college/university	1 (25.0%)	2 (18.2%)
- Bachelor’s Degree	2 (50.0%)	(0.0%)
**Health care professionals**		
**Characteristics**	**Australia (*n*** **= 6)**	**Hong Kong (n = 11)**
**Age in years n (%):**		
- 21–30	4 (22.2%)	0 (0.0%)
- 31–40	7 (38.9%)	6 (54.5%)
- 41–50	4 (22.2%)	0 (0.0%)
- 51–60	3 (16.7%)	5 (45.5%)
**Gender n (%):**		
- Female	17 (94.4)	7 (63.6%)
- Male	1 (5.6%)	4 (36.4%)
**Discipline n (%):**		
- Nurse	13 (72.2%)	6 (54.5%)
- Dietitian	4 (22.2%)	2 (18.2%)
- Doctor	1 (5.6%)	3 (27.3%)
**Employment status n (%):**		
- Full time	7 (38.9%)	11 (100.0%)
- Part time	11 (61.1%)	0 (0.0%)
**Highest level of education qualification n (%):**		
- Doctoral Degree	1 (5.6%)	5 (45.5%)
- Master’s Degree	3 (16.7%)	0 (0.0%)
- Post graduate speciality qualification	2 (11.1%)	1 (9.1%)
- Bachelor’s Degree	10 (55.6%)	1 (9.1%)
- Diploma	2 (11.1%)	1 (9.1%)
- Specialist training	0 (0.0%)	3 (27.2%)
**Clinical experience in years n (%):**		
- ≤ 5	5 (27.8%)	0 (0.0%)
- 6–10	4 (22.2%)	2 (18.2%)
- 11–15	5 (27.8%)	1 (9.1%)
- > 15	4 (22.2%)	8 (72.7%)
**Involvement with the care of a patient in the (intervention name blinded for peer review) study or their family member n (%):**		
- Yes	7 (38.9%)	1 (9.1%)
- No	7 (38.9%)	9 (81.8%)
- Unsure	0 (0.0%)	1(9.1%)

AUD – Australian dollars; HKD – Hong Kong dollars; IQR – interquartile range; MST – malnutrition screening tool; PG-SGA SF – Patient-generated Subjective Global Assessment Short Form

Participants expressed their perceptions of, and experiences with, participating in the PIcNIC intervention, in their capacity as a patient or family member receiving the intervention, or as a staff member delivering the intervention, caring for a patient who had received it, or providing opinions although not directly exposed to the intervention. Their responses formed two categories, which are described below. The redundant concepts for category 1 were: the intervention works in outpatient settings, the food diary is easy but needs to be tailored, the information booklet is a good resource, and the intervention should be delivered by a dietitian, but could be delivered by a nurse. The redundant concepts for category 2 were: a personalised nutrition plan is required, patient and family involvement in the intervention is valued and the intervention has benefits for patients and families. Converging and diverging perceptions of each redundant concept are presented below.

### Category 1: context and intervention feasibility and acceptability

Our analysis shows that the intervention is most feasible and acceptable when delivered in the outpatient setting, by a dietitian, using the current information booklet and a modified version of the food diary (Table 4). Descriptions of the redundant concepts follow.

**Table 4:** Convergence and Divergence in feasibility and acceptability of the intervention and context

Redundant concepts	Australia Patient	Australia Family	Australia Health Care Provider	Hong Kong Patient	Hong Kong Family	Hong Kong Health Care Provider	Reason for divergence
The intervention works in outpatient settings	C	C	C	C	C	C	N/A
The food diary is easy but needs to be tailored	C	C	D	C	C	D	The food diary is burdensome and does not support context-specific food choice
The information booklet is a good resource	C	C	D	C	C	D	HCPs were impartial to the value of the booklet.
The intervention should be delivered by a dietitian^a^, but could be delivered by a nurse	N/A	N/A	C	N/A	N/A	C	N/A

^a^Interview questions related to who should deliver the intervention were only asked of HCPs

*C* convergence, *D* divergence

#### The intervention works in outpatient settings

The context in which the intervention was delivered influenced perceptions of intervention feasibility. For example, patients, families and health care professionals all noted that delivering the intervention in the inpatient setting was challenging for three key reasons. The first was that while in hospital, patients either felt too unwell to be able to participate in a meaningful way or, in some cases, patients were admitted for a short period of time (1–2 days) which made intervention delivery challenging:

*‘It (education session) was good but I think I was a little bit tired, which I still am, and I just want it over and done with, so I could go to sleep. Sweetheart, I’m an old lady, I’m 72 years of age, maybe for younger people yes, but for me as I said, since I’ve been diagnosed all I want to do is sleep.’ Inpatient 1, Australia.*

Conversely in the outpatient and home setting, patients may be in better condition to receive the intervention: *‘In outpatient or in home-setting I find … patient’s condition is quite better … [they] have energy to take this information.’ (Home Care Nurse, Focus Group 1, Hong Kong).*

The second was that family engagement in the inpatient context was likewise challenging as, unlike outpatient- or community-based intervention delivery, dietitians could not set a specific time for intervention delivery because of competing work demands. Family members sometimes did not visit or tended to visit later in the afternoon or evening, after the dietitian had left for the day, which limited their involvement: “*… it’s sixty-forty, sixty percent of families will have lots of family support and be very involved and forty percent you mightn’t even see anybody on the ward so it really just depends …*” *(Senior Nurse 1, Australia).* Conversely in the outpatient department (Australia) or community setting (Hong Kong) it was easier to engage family or home carers in the intervention because specific times were set for consultation with the dietitian:

“*… at the end of the day the majority of cancer patients are treated in the outpatient setting and we have thirty inpatients out of thousands on the ward at any one time … I think we get the biggest bang for out buck when we target interventions in the out-patient setting; the inpatient stays aren’t a part of everyone’s journey … we really need to be looking at the most appropriate setting for engaging family members and I believe that’s the out-patient setting.” Dietitian 1, Australia.*

Third, nutritional goal setting, which was a feature of the intervention, was difficult for patients and families to enact in an inpatient setting where food choice was limited, and food options offered were inconsistent with patient preferences and/or symptoms: *“If you have a lot of nausea … they don’t have any plain mineral water. They don’t have any ginger beer and all those things help people with nausea.” (Inpatient 2, Australia).* Hospital systems, such as scheduled mealtimes, and rules which prevented re-heating food or bringing food in from home limited how patients could enact aspects of the intervention. These restrictions also made nurses ‘*feel helpless’ (Nurse, Focus Group 1, Australia)* as they wanted to encourage interventional elements but were unable to provide patients with flexible food options for their needs.

#### The food diary is easy but needs to be tailored

With reference to specific intervention components, namely the food diary, perspectives of patients and families differed from that of health professionals. Patients and families found the food diary easy to complete, described as *“It’s not difficult to record.” (Outpatient 1, Hong Kong)* and most were confident in their ability to assess intake and record this in the food diary.

However, these participants did request modifications. In Australia, participants requested more space for additional items: *“I actually had water or a cordial extra … and even though that’s minor, it’s in my day … probably more area for fluid intake because I don’t just have water. I have cordial or tea and coffee.” (Inpatient 3, Australia).* In Hong Kong some participants had preferred to write out foods on a blank form, because the pre-selected items did not accommodate their range of food choices: “*… it all depended on the patient’s family diet habit. If the family did Chinese cooking, it [food diary] would be useful.” (Family member 1, Hong Kong).*

Conversely, health professionals perceived the food diary to be too complex, lengthy, difficult and burdensome for families, leading them to believe completion rates would be low. They also questioned the value of information provided in the food diaries given that it was not able to accommodate a full range of food choices and lacked portion size estimates:

*“this record sheet seems to be a bit lengthy … actually quite difficult for them to go through. For usual patients or some usual caregivers in our district. Most of them might not have that kind of intelligence in filling [the form] … it really depends on the educational level … There may be some potential difficulties because in this written record usually is very difficult to quantify the amount he is taking.” (Doctor 1, Focus Group 1, Hong Kong).*

#### The information booklet is a good resource

Dissimilar perspectives were observed in relation to the nutrition booklet, another specific intervention component. Patients and families described the nutrition booklet as having *‘aspects of nutrition information [that] were useful’ (Family member 2, Hong Kong),* and *‘ … something you can always read back on if you need to get some more information later on.’ (Inpatient 4, Australia):*

*“It was very informative actually. Yes. I understand a little bit better how to eat. I was eating proper I thought, but no. It’s helped me a lot.” (Inpatient 3, Australia).*

Overall, health care professionals were neutral about the value of the education resource and provided more specific printed material to the patient to supplement information provided in the nutrition booklet. Some dietitians placed more value on their verbal interaction with the patient and family.

#### The intervention should be delivered by a dietitian, but could be delivered by a nurse

In the context of this study, the intervention was able to be delivered as planned. There was general agreement amongst health professionals that the intervention would be best delivered by a dietitian. However, in the Hong Kong setting, home care nurses were best positioned to deliver the intervention as they already visited patients in their home regularly, had the knowledge and skills to talk to patients and families about nutrition, were consistent across the continuum of care, and had existing relationships with patients and families:

*“If it is outpatient, I think the home care nurse will be the best person, if they are equipped with enough background knowledge … they will go there every time, many times during the whole trajectory of the patient … I think talking about eating, talking about diet, is one thing that can engage patient and family in it.” (Doctor 2, Hong Kong).*

Participants recognised that nurses may require additional nutrition-related training from dietitians to ensure delivery of consistent messages and needed to be able to refer more complex cases to an outpatient dietitian:

*“I think that a dietitian can teach us some simple assessment tools and education to give the patients and caregivers. For the complex cases, we can refer the patients to the dietitian for further intervention or advice.” DOM Nurse, Focus Group 2, Hong Kong.*

Conversely, in Australia, dietetic care was available for both inpatients and outpatients, and it was suggested that “*… it could be the dietitian or the nutrition or allied health assistants that deliver that initial intervention.” (Dietitian 2, Australia).* Although in the inpatient setting, dietetic resources were scarcer and delivery of the intervention in partnership with a nurse was considered potentially beneficial.

### Category 2: benefits of patient- and family-centred nutrition care

Our analysis demonstrates that the intervention enhanced patient-and family-centred care by enabling individualised nutrition planning and patient and family involvement in care; and provided benefits to patients and families (Table 5).

**Table 5:** Convergence and Divergence in benefits of patient- and family centred nutrition

Redundant concepts	Australia Patient	Australia Family	Australia Health Care Provider	Hong Kong Patient	Hong Kong Family	Hong Kong Health Care Provider	Reason for divergence
An individualised approach to nutrition planning is required	C	C	C	C	C	C	N/A
Patient and family involvement in the intervention is valued	C	C	C	D	D	D	The intervention may result in family conflict
The intervention has benefits for patients and families	C	C	C	C	D	C	Some families maintain food myths

*C* convergence, *D* divergence

#### A personalised nutrition plan is required

Tailoring of nutrition care by the person delivering the intervention was valued by all groups across all settings. Patient and family participants believed that nutrition education and goals should be tailored to the patient and liked the fact that the intervention allowed for adaptation to patients’ dietary preferences and habits, consistent with their background or culture. Intervention acceptability was exemplified in a comment from one patient who expressed feeling more *‘comfortable’* because the intervention was *‘made just for me’ (Outpatient 4, Australia)*. Others appreciated the follow-up after the initial education session, describing this as an opportunity to discuss any *‘problems with meals’ (Outpatient 2, Hong Kong)* and to ask any clarifying questions. This view was supported by health care professionals who believed nutrition education and interventions should be tailored to patients’ and families’ needs, values, disease stage, dietary habits and preferences.

*‘We really need to assess them [the patient] individually, based on what is the [disease] stage and what their current eating habits [are] … .before you can implement and make [a] suggestion … there’s no formula that fits everyone … .’ (Doctor 2, Hong Kong).*

In Hong Kong, it was suggested that tailoring of the intervention should incorporate beliefs about foods. They explained that in Chinese culture, people may have *‘strange’ (Doctor 2, Hong Kong)* beliefs about food and link eating with prognosis and survival; and the taste of food may be more important than nutritional value.

#### Patient and family involvement in the intervention is valued

Patients reported increased feelings of support, encouragement and assistance with family/ caregiver involvement. As one patient commented, *“It’s just support and, you know, not feeling as if you’re on your own and that you’ve got a big job to do - that you can share it with somebody.” (Outpatient 10, Australia).* Families helped with remembering information during the dietitian consultation and implementing positive nutrition practice at home. Several patients said they relied heavily on their family members for nutrition, as the family had been *‘cooking food and bringing (me) snacks’* and that they *‘wouldn’t eat’* without the family member’s assistance. Families were also able to support the patient during the education sessions by re-wording difficult phrases or extracting more detailed food recalls from patients to provide more comprehensive nutrition intake information. “*… I was asking a lot of questions, because my husband really couldn’t remember from one minute to the next. … I know he wasn’t eating because the ladies who bring the trays in would say he hadn’t touched it.” (Family member 1, Australia).*

Health care professionals also valued family participation, acknowledging the important insights family members brought to the nutrition consultation, emphasising that families *‘know the patient much more than health care professionals, or even dietitians. So, it’s easy for them to choose the kinds of foods the patient would like … according to their culture, or according to patient preference … ’ (Nurse Consultant 1, Focus Group 1, Hong Kong)*. Including patients and family members as active participants in discussions about specific foods and nutrition goals was viewed as important by dietitians, as they thought personalised nutrition care would *‘help the patient longer term’* by allowing them to understand the reasoning behind food suggestions, and that this would lead to more sustainable health practices (Dietitians 1 & 2, Australia). Further, involvement of family caregivers was seen to increase provision of accurate information (such as the diet history) in sessions where family were present, and nutrition information delivered to family was seen to increase the likelihood of recommendations being implemented at home.

*‘We need the input about the set goal for that family. Because they know the preference about the patient. They know the tolerance of diet. They take care [of] the patient daily, they know everything about the patient. Maybe they [can be] involved in the setting of goal. Not … calories and protein, but. … to choose what kind of food, the meal pattern, and the whole planning.’* (*Dietitian 1, Focus Group 1, Hong Kong).*

There were some negative perceptions to family participation in Hong Kong. Families reported inability to achieve their nutrition goals, most commonly due to patient fullness, and in turn patients reported declining food and/or being forced to eat, which could result in conflict: *“The nutritional plan was to have small frequent meals; sometimes it was possible to follow the plan but not when my mum was full.” (Family member 3, Hong Kong).* Similarly, Hong Kong health providers commented that families could become very distressed when the patient was not eating well, which could result in them not listening to patients’ wishes (e.g. force-feeding patients), causing unwanted conflict between patient and family: “*… sometimes conflict with the patient. They want to push the patient to eat more, get more nutrition, get better, but the patient says, “no I have lost appetite, I don’t want to take this.” (Ward nurse 1, focus group 1, Hong Kong).* Finally, some patients in Hong Kong had domestic carers and health care professionals questioned their ability to provide the same benefits as family engagement in the intervention.

#### The intervention has benefits for patients and families

Learning was the main outcome perceived from patients and families in this study. At both sites, patients and families learned about the importance of protein for cancer patients: “*… it pointed me in the right direction as far as high proteins … it was really good to refresh my memory as to what was important nutrition wise.” (Inpatient 5, Australia).* Australian patients/families reported broad outcomes like new perspectives and heightened awareness around nutrition. In Hong Kong patients/families spoke about more specific learning around food choices, the importance of small frequent meals, portion sizes and food preparation for symptom management: *“Before meeting with the dietitian, I misunderstood I could eat 4 tael [Chinese weight system] of meat per meal; I therefore ate a total of 12 tael of meat for 3 meals until the dietitian explained, I should separate the 4 tael of meat into 3 meals instead.” (Outpatient 3, Hong Kong).* Many Hong Kong families stated after learning, they changed their food practices and choices to encourage dietary intake, and this was perceived to increase patients’ intakes and weight as a result.

In addition, myths about foods perceived to either be beneficial or harmful for patients with cancer were dispelled. Family members/caregivers reported an increase in nutrition knowledge and confidence in selecting foods following the dietitian consultation. In turn, relationships were also strengthened when patients and families addressed nutrition care as a team, with the wife of one patient stating they were *‘agreeing a lot more [on nutrition]’ (Outpatient 14, Australia)*. Several participants said they had been including a larger variety of foods in their diet since being involved in the intervention and had introduced *‘all kinds of foods’ (Outpatient 8, Australia)* that were previously perceived to be off-limits: *“one relative commented that that’s very good … because she knows how to make some different food for the patient. And she was surprised that the dietitian taught her to give … Horlick and sandwiches to the patient, she’s surprised that she can try to give this, not just only rice, or congee.” (Nurse 1, Focus Group 2, Hong Kong).* Patients described the added support family members could provide when they were challenged by well-meaning people to adhere to a diet that aligned with cancer food myths such as, believing certain diets such as Gerson, alkaline or ketogenic diets cure cancer, because their family member had similar nutrition knowledge from attending the intervention. However, in Hong Kong, some families still reported diet-induced conflict and continued to hold their preconceived ideas about what foods were off-limits for patients with cancer.

Staff perceived many benefits for families participating in the intervention. Families were seen by health professionals to be *‘more informed’* as it *“brings to the forefront that dietary requirements are important”* and in turn, families made nutrition decisions based on the *‘right information’* after attending the sessions (Nurse, Focus Group 2, Australia). Staff thought *‘giving family members something to do’ (Doctor 2, Hong Kong)* around nutrition served to manage distress experienced by family members of a patient diagnosed with cancer: “*… eating” also not only for the food, also for the whole family, not only for the physical symptoms and also [to] treat the psychological. Because the family thinks that if [the patient] cannot eat, maybe the survival time is decreased.” (Nurse 2, Focus Group 2, Hong Kong).* For many, this was seen as the most important outcome – even more than actually increasing nutrition intakes:

*‘Our goal is not [to] emphasize too much about calories and protein, but we realized … many family members are quite stressful [sic] about preparing food … . So, I think nutritional advice can help them to choose foods that are tolerated by the patient that … so they can enjoy the food … that can help to release the tension … relieve the pressure of the both family and patients.’ (Dietitian 1, Focus Group 1, Hong Kong).*

Likewise, family members highly valued the dietitian teaching them how to prepare different foods for patients to tolerate and enjoy, which helped manage symptoms and enabled them to meet their needs and preferences. This learning provided a role for family members which reduced stress for both the family and the patient: *“In fact, your study, your support is kind of psychological support more than physical support to the patient … this is how I would describe our present family situation”* (*Family Member 4, Hong Kong).*

## Discussion

In this study, the PIcNIC intervention was demonstrated as acceptable when delivered in the outpatient or home setting, with tailored verbal information provided by a dietitian (or possibly a nurse) and supporting written material provided. In our study, we found that both patients and families valued receiving nutrition-specific information that addressed knowledge deficits and assisted them in making choices about foods and strategies to help support nutrition intake and minimise nutrition impacting symptoms [14]. Patients and families valued both passive (written nutrition booklet) and active (health care professional education and reinforcement) nutrition-specific information dissemination strategies. This resonates with systematic review findings on disseminating recommendations to patients, whereby combing passive and active strategies is most effective to improve health [15]. Of particular value to our participants was that verbal information provided by the dietitian was tailored to the patient’s diagnosis, nutrition impacting symptoms, and personal preferences, with information provision guided by questions generated by the patient and family. Patients and families may have respected this educational approach because it was provided by a well-regarded health care professional [15] and was individualised, which influences patient satisfaction [16]. General nutrition-related information provided in written resources was consistent with recommendations for passive resources, as it was viewed as clear, specific, in lay-language, and able to be referred back to [15]. Overall, the education dissemination approach was consistent with expert recommendations specific to cancer-related malnutrition [1], as nutrition information following a cancer diagnosis is essential to inform patients about strategies that can help to optimise weight, reduce nutrition impacting symptoms and improve nutrition literacy [17], and information was tailored to patients’ estimated energy expenditure, disease state, current intake, lifestyle and food preferences.

For Australian participants, explanations around cancer food myths such as ‘sugar fuels cancer’ and the notion that ‘milk and milk products enhance breast cancer growth’ were welcomed and helped participants make informed choices about their food selections. Importantly, it provided participants with knowledge and sense of empowerment to help them manage the plethora of advice provided by well-meaning friends and family. Education provided to families focused on having a high protein, high energy and well-balanced diet rich in vegetables, fruits and whole grains, and limited in red meat and alcohol [18]. While cancer-related food myths were not as prevalent among the Hong Kong participants, philosophical and religious views are recognised as factors which influence health-related beliefs of the Chinese [19]. Specifically, diet and food choices are believed to be important to the yin-yang concept an important in maintaining balance and therefore health [20].

Documentation of nutrition intakes through the food diary was generally well accepted by patients and families. However, health providers did not necessarily see the value in these and believed that they were difficult to use while also not providing detailed information about nutrition intakes. Traditionally, nutrition diaries are used as a method of quantifying nutrition intake for the purpose of determining protein and energy intakes. However, in this study the intent of the diary was to serve as a prompt for monitoring nutrition intakes and having discussions about strategies to improve intakes and minimise nutrition impacting symptoms. The format of the diary could be improved by being less prescriptive and allowing greater flexibility to allow for documentation of a range of foods was seen as important, particularly when patients were at home and exposed to a wider range of foods that was normally provided in the hospital.

The PIcNIC intervention may facilitate patient and family centred care, by promoting patient and family involvement in nutrition care, while influencing perceived outcomes like increased learning for patients and families and providing families with purpose. We intentionally adopted a patient- and family-centred approach to delivering this intervention, which is consistent with recommendations from international health organisations and is driven by the growing body of evidence that demonstrates improved outcomes associated with patient-centred practices [21–23]. Increasingly, patient-centred nutrition interventions are being developed in the context of both acute [24–27] and chronic health conditions [28]. In this study, participants perceived increased patient and family engagement in nutrition care, a key feature of patient and family centred care [29]. As demonstrated in a recent systematic review by Sladdin et al. (2017), there is a strong desire by patients to be involved with and participate in their nutrition care. In turn patient and family involvement in nutrition care may improve outcomes. For instance, Kim et al. [30] found that high patient involvement in the co-creation of personalised dietary plans resulted in improved dietary intake and functional status among patients, as well as increased patient satisfaction. Further, an intervention by Sathiaraj et al. [31] described similar benefits from patient participation in their nutrition care, with increased energy and protein intakes, body weight, and high patient satisfaction apparent when family members and patients were highly involved in dietary decisions.

The intervention may also enhance food enjoyment, an outcome that can be fundamental in cancer patients, linked with social, cultural and family aspects of life. Families were also included in the current study because they often provide support to the patient across a range of areas, including dietary practice [32]. The impact of a cancer diagnosis on the family can also be considerable, and it has been reported that family caregivers can often feel more anxious about weight loss and poor appetite than the patients themselves [33]. Collectively, these findings indicate that the purposeful inclusion of patients and family caregivers in the patient’s nutrition care and decision-making processes can result in improved nutrition- and patient-related outcomes.

Most family members felt that joint participation in the intervention strengthened their relationship, as patients perceived feeling supported by their family member in nutrition decisions and approaches to nutrition care. However, some Hong Kong participants did report conflict when patients had early satiety and families continued to encourage intake, although it is possible that such conflict could possibly arise, irrespective of the intervention. Macmillan Approach to Weight and Eating studies conducted in the United Kingdom suggest that while family member influence can be helpful, it can also be detrimental to quality of life and the patient’s nutritional intake because disagreements existed about food and eating [34]. The disagreements were typically fuelled by uncertainty about what was best for the patient. As our approach specifically targeted patients and their family member, it is possible that uncertainty was mitigated by ensuring a consistent message was delivered to the family unit.

While the intervention was well received by patients, families and health care professionals, undertaking this study in inpatient and outpatient contexts in two countries contributed to learnings about intervention feasibility. The ability of patients and family members to actively participate in the intervention were identified as a factor which influenced intervention feasibility Challenges in engaging families in nutrition care in an acute care context has been previously reported in the intensive care unit where 45.7% of families were not enrolled in the study because of family dynamics (8%), consent declined (20.3%) or the family member was not contactable (17.4%) [7]. Incorporating intervention delivery into a regularly scheduled outpatient appointment and encouraging families to be present at this appointment improved family participation. However, not all patients had family members present. In some instances, this was because the family member was not able to attend, and in others because the patient chose to participate in the intervention alone.

Our data suggest that a patient- and family-centred nutrition intervention in the context of cancer care is best delivered in an outpatient setting. While dietitians are best placed to provide intensive, individualised nutrition counselling to people with cancer [35], this may be challenging to enact in some contexts where there is a low dietitian-to-patient staffing ratio. Internationally, staffing levels fluctuate widely across countries and clinical contexts [36, 37] as clear recommendations for staffing levels are lacking. This suggests that alternate strategies for intervention delivery may be required. In the context of the Macmillan Approach to Weight Loss and Eating difficulties, it was reported that nurse-delivered nutrition information was acceptable to patients and this approach resulted in a slower decline in performance status in cancer patients [38]. However, with only 39% of surveyed nurses considering their basic nutrition education sufficient [39], there is a need to improve nurses’ education in relation to nutrition care [40]. This concern was also highlighted by the health care professionals in our interviews. These challenges may signal the need for an interdisciplinary approach to nutrition care for patients with cancer, with dietitians and nurses working collaboratively with patients and their families to monitor nutrition status and implement tailored nutrition plans to optimise nutrition-related outcomes [41, 42].

### Limitations

A strength of this study was the multiple contexts in which the intervention was evaluated. Having data from two different countries and through including hospitalised patients, outpatients seen in hospital clinics, and outpatients receiving home-based care we were able to develop a stronger understanding of varied contexts which may influence the successful implementation of the intervention. It is possible the intervention fidelity varied across sites and we did not collect detailed information regarding how the intervention was actually delivered. Testing of the PIcNIC intervention will be required in future research and at this time a stronger focus on intervention fidelity is recommended.

We collected data from various participants to develop a broad understanding of perspectives of patients, their families (and paid carers), and a range of health professionals. Although many health care professionals who were interviewed, were not exposed to the PIcNIC intervention, they were recruited from settings where the intervention would be applicable and had in-depth knowledge of these patients and families from these settings, they were able to provide valuable insights into intervention acceptability.

An interpretive approach was undertaken for analysis, which may be viewed as a limitation. However, the use of more than one researcher to analyse findings, with constant input from a third researcher to question and confirm findings increases the credibility of our findings. In addition, the inclusion of divergent views and a wide range of perspectives on the topic (patients, family and health care professionals) establishes the confirmability of findings.

Nutrition risk was higher in the participants from the Hong Kong study site. Whether this influences receptiveness to the intervention is unclear. In future studies evaluating intervention effectiveness attention will need to be given to selection of patients at increased nutrition risk who are likely to confer greater benefit from a nutrition intervention.

## Conclusions

Our intervention specifically focussed on creating a patient- and family-centred approach towards nutrition education, and our acceptability data suggests we fostered patient-and family centred partnerships with health professionals. From this preliminary data we can see that participants view the intervention favourably owing to combined methods of information dissemination including tailored health care professional input, written material, and reinforcement which may increase perceived learning. The effectiveness of future interventions that involve patient and family partnership is contingent on setting, this intervention worked best when delivered in outpatient and home care setting. The availability of families and well-positioned health care professionals in these settings may allow health care professionals to more readily incorporate patient and family centred care into their clinical practice.

## Supplementary information

**Additional file 1.**

## Data Availability

The datasets generated and/or analysed during the current study are not publicly available because personal confidentiality may be compromised, data and materials are available from the corresponding author on reasonable request.

## Abbreviation

PIcNIC: **P**artnering with fam**i**lies to promote **n**utrition **i**n **c**ancer care
